# The interface of psychiatry and COVID-19: Challenges for management of psychiatric patients

**DOI:** 10.12669/pjms.36.5.3073

**Published:** 2020

**Authors:** Afzal Javed, E. Mohandas, Avinash De Sousa

**Affiliations:** 1 Afzal Javed Immediate Past President - Asian Federation of Psychiatric Associations & President Elect-World Psychiatric Association & Consultant Psychiatrist & Chairman, Pakistan Psychiatric Research Centre, Fountain House, Lahore, Pakistan; 2 E. Mohandas Member, Pharmaco-Psychiatry Section, World Psychiatric Association and Past President - Indian Psychiatric Society and Consultant Psychiatrist, Sun Medical and Research Centre, Trichur, Kerala, India; 3 Avinash De Sousa Consultant Psychiatrist and Research Associate, Department of Psychiatry, Lokmanya Tilak Municipal Medical College, Mumbai, India

**Keywords:** COVID, psychiatric medication, pandemic, telepsychiatry, telemedicine

## Abstract

COVID-19 is a viral infection that has multisystemic physical and psychological complications. The following paper looks at the various challenges seen while treating psychiatric patients during the COVID pandemic. There is a need for physician to be aware of the drug interactions between psychiatric medications and the medications used routinely in the management of COVID. There is also the concern of psychiatric side effects of medications used to manage COVID and medical complications caused by some side effects of psychiatric drugs. The telepsychiatry and telemedicine paradigm has made it mandatory for physicians to be vigilant of the same.

The COVID-19 pandemic has brought new challenges for the management of psychiatric patients and the administration of psychiatric treatments. The interface of psychiatry and COVID-19 is relevant for both COVID positive and negative cases.^1^ The COVID-19 pandemic has resulted in a surge of patients with first episode of psychiatric disorders like depression, anxiety and even acute psychosis. There have also been reports of acute exacerbation and relapses of patients with pre-existing psychiatric diagnoses.^2^ Patients with psychiatric disorders must adhere to their medications and be compliant on the same. The ensuing lockdown in many countries has opened up vistas of teleconsultation and various agencies have proposed telepsychiatry guidelines.^3^ Even though prescriptions are issued via teleconsultation, there is a need for monitoring and managing medication induced adverse effects.

COVID-19 is a multisystem disorder that affects the liver, kidneys, lungs and heart, as well as the immune and hematological systems. This could lead to pharmacokinetic changes that impact absorption, distribution, metabolism and/or excretion of psychotropic medications and increased propensity to develop adverse effects. Clinicians treating patients with COVID infection must be aware of the need to make adjustments to existing psychotropic drug regimens and/or avoid using certain psychotropic agents if needed.^4^,^5^ Many psychiatric medications including sodium valproate, carbamazepine, tricyclic antidepressants, antidepressants like serotonin norepinephrine reuptake inhibitors (SNRIs) and atypical antipsychotics have been associated with mild hepatoxicity with elevated liver enzymes on testing. Few psychiatric medications like carbamazepine, duloxetine and chlorpromazine may also have serious liver toxicity. These should be preferentially avoided in patients with COVID-19 associated liver disease.^6^

COVID-19 infection can cause hematological problems (lymphopenia, thrombocytopenia and disseminated intravascular coagulation). Special care has to be exercised while prescribing clozapine, SSRIs and high dose of valproate.^7^ If there is moderate to severe renal impairment it is better to avoid psychotropics highly dependent on renal excretion like lithium, gabapentin, topiramate, pregabalin, duloxetine and paliperidone.^8^ Similarly COVID-19 can produce myocarditis and arrhythmias so it would be better to avoid psychotropics with a propensity to prolong QT interval.^9^

In COVID-19 patients with delirium, clinicians should be very careful when prescribing benzodiazepines, opioids, drugs like tricyclic antidepressants and low potency antipsychotics as they may cause or exacerbate confusion, sedation and/or falls. One must be cautious about prescribing psychotropics that can lower the seizure threshold in patients with seizures or structural brain lesions and cause drug induced seizures which may complicate the existing medical problems further.^10^

One must also be vigilant about psychiatric side effects that may come up with corticosteroid use in COVID patients. These include depression, mania, agitation, mood lability, anxiety, insomnia, catatonia and psychosis. The majority of these side effects occur early in treatment, within days and is usually seen at high doses (Prednisolone equivalents of >40mg/day).^11^ Other important tasks for the psychiatrist treating a COVID-19 patient includes a complete review of all medications, monitoring psychiatric side effects of all medications and differentiating between primary psychiatric symptoms versus those that are secondary to COVID-19 or other medications.

Clozapine is the most effective antipsychotic used for reducing positive symptoms, management of treatment resistant schizophrenia and many patients in centers around the world are maintained and symptom free on Clozapine. The drug needs monitoring along with absolute neutrophil count (ANC) monitoring which may be difficult due to the movement restrictions and lockdown caused by the pandemic.^12^ It may be essential to continue Clozapine as an abrupt stoppage of the drug causes relapse or exacerbation of severity of existing illness which is wrong in a patient that was well maintained on the drug. It has been recommended that “the frequency of ANC monitoring may be reduced to every three months, with dispensation of up to a 90-day supply for people fulfilling all of the following criteria viz. continuous clozapine treatment for > 1 year, having never had an ANC < 2000/µL (or < 1500/µL if history of benign ethnic neutropenia) and with no safe or practical access to ANC testing.”^13^ For patients on clozapine with any symptoms of infection (including those reported for severe acute respiratory syndrome coronavirus 2 [SARSCoV-2], such as cough, fever and chills, sore throat or other flu-like symptoms), an urgent physician assessment including a complete blood count (with ANC) should be obtained. The clinical assessment could take place either in person or by telehealth based on local protocols.^13^,^14^

If patients on clozapine become symptomatic with fever and flu-like symptoms, “the emergence of signs and symptoms of clozapine toxicity may require clinicians to reduce the dose of clozapine by as much as a half. Continue the lower dose until three days after the fever has subsided, then increase clozapine in a stepwise manner to the pre-fever dose.” Where available, clozapine levels help facilitate clinical decision-making, particularly after substantial dosage change, inadequate response or unexpected adverse effects.^13^,^14^

Hydroxychloroquine is recommended by some researchers as treatment in Covid 19 based on its mechanism of action: increases endosomal/lysosomal pH and inhibits viral replication, inhibits major histocompatibility complex class II expression, antigen presentation and immune activation (reducing CD154 expression by T cells) via Toll-like receptor signaling, interferes with cyclic GMP- AMP (cGAMP) synthase (cGAS) activity and reduces the production of various pro-inflammatory cytokines. Chloroquine and hydroxychloroquine can rarely produce behavioral side effects, lightheadedness, sleep disturbances, irritability, confusional states and psychosis.^15^,^16^

CYP enzymes catalyse the dealkylation of chloroquine and hydroxychloroquine to pharmacologically active metabolites. CYP2C8, CYP3A4, CYP2D6 and CYP1A1 can metabolize chloroquine. Chloroquine or its analogues when used with psychotropics with CYP3A4 inhibitory potential, side effects will ensue due to raised levels of chloroquine. CYP3A4 inducers could decrease levels of chloroquine or hydroxychloroquine. The combined cardiac side effects of chloroquine analogues must be kept in mind when prescribed in conjunction with psychiatric medications that prolong QTc interval with the resultant cumulative cardiac toxicity. In addition, aminoquinolines decrease activity of immunosuppressants and antibiotics.^17^

Antiviral agents with probable beneficial effects include remdesivir, lopinavir/ritonavir, lopinavir/ritonavir combined with interferon-β, convalescent plasma, and monoclonal antibodies. Remdesivir can increase hepatic enzymes. Hypotension, diaphoresis and shivering associated with remdesivir should not be mistaken as a panic attack. In vivo studies on pharmacokinetic properties of remdesivir are lacking.^18^

Ritonavir was found to be a potent inhibitor of CYP3A-mediated biotransformation. Ritonavir is also an inducer of CYP1A4, glucuronosyl transferase (GT), and possibly CYP2C9 and CYP2C19. Agents that increase CYP3A activity, such as carbamazepine, phenobarbital and phenytoin, increase ritonavir clearance, resulting in decreased ritonavir plasma levels. Alprazolam, diazepam, estazolam, flurazepam, midazolam, triazolam, and zolpidem may cause extreme sedation and respiratory depression when used together. Bupropion and clozapine when used together with ritonavir may increase plasma levels of these drugs, thus increasing the patient’s risk of arrhythmias, hematologic abnormalities, seizures, or other potentially serious adverse effects. Ritonavir formulations contain alcohol that can produce disulfiram like reactions when used with metronidazole or disulfiram. Ritonvir may increase statin levels in patients as well.^19^,^20^

Colchicine, a plant-derived alkaloid with anti-inflammatory properties, is metabolized by CYP3A4 and excreted via the P-glycoprotein (P-gp) transport system. Dose adjustment is recommended with concurrent use of CYP 3A4 or P-gp inhibitors as well as in patients with hepatic or renal impairment. Ivermectin metabolized in liver by CYP3A4 isoenzyme is a potent inhibitor of P-glycoprotein. Ivermectin may increase prothrombin time necessitating caution when used with warfarin.^21^,^22^

Chloroquine, hydroxychloroquine sulfate (HCQS) and antiretrovirals are the mainstay for the treatment of COVID-19 infection.^23^ While these drugs may have modest to good efficacy in the management of COVID it is vital that treating physicians be aware of various treatment emergent side effects of a psychiatric nature that may be seen with these drugs.

Chloroquine is a drug which has been used in the treatment of malaria since the 1960s. There have been many anecdotal case reports and case series of chloroquine induced psychosis when the drug has been used as an antimalarial and the same shall hold true even when the drug is used in the management of COVID. This has been attributed to probable iatrogenic and probable mechanisms via the pharmacogenetic and cytochrome mechanisms.^24^-^26^ Clinicians will have to be vigilant for the emergence of drug induced psychosis with the drug. There have also been anecdotal reports of chloroquine induced mania in patients treated with the drug for malaria.^27^ The development of these side effects would warrant withdrawal of the drug and treatment with antipsychotics, while affecting the management of COVID in these patients.

Hydroxycholorquine sulfate has also been implicated in the development of anxiety, depression and acute psychosis in patients with rheumatoid arthritis treated with drug.^28^,^29^ Thus physicians have to be aware of the same and be vigilant when treating patients with COVID. Antiretroviral drugs used in the management of COVID via their action on the immune system may cause depression as a side effect in many patients.^30^ Data on the efficacy and safety as well as side effect profile of drugs like Remedesivir used in COVID are still scare but nevertheless one has to monitor for side effects that may ensue.^31^ It is also essential for clinicians to be aware that symptoms of a neuropsychiatric nature like those mentioned above in patients with COVID may not always be drug induced and rather can also be caused by the huge cytokine storm due to the disease process and neuroinflammatory mechanisms.^32^

The ensuing lockdown in many countries has opened up vistas of teleconsultation and telepsychiatry guidelines with respect to prescribing have been developed by various agencies. Even while prescriptions are issued via teleconsultation, there is a need for monitoring side effects and protecting the patient from any adverse effects that may be medication induced. There are many areas in psychiatric treatment where caution has to exerted during the COVID pandemic.^33^ Vigilance must be exerted by medical specialists and psychiatrists alike when handling patients during the current scenario.

## References

[1] Duan L, Zhu G (2020). Psychological interventions for people affected by the COVID-19 epidemic. Lancet Psychiatry.

[2] Haider I, Tiwana F, Tahir S (2020). Impact of the COVID-19 Pandemic on Adult Mental Health. Pak J Med Sci.

[3] Hollander JE, Carr BG (2020). Virtually perfect?Telemedicine for COVID-19. New Engl J Med.

[4] Bilbul M, Paparone P, Kim AM, Mutalik S, Ernst CL (2020). Psychopharmacology of COVID-19. Psychosomatics.

[5] Wang T, Du Z, Zhu F, Cao Z, An Y, Gao Y (2020). Comorbidities and multi-organ injuries in the treatment of COVID-19. Lancet.

[6] Telles-Correia D, Barbosa A, Cortez-Pinto H, Campos C, Rocha NBF, Machado S (2017). Psychotropic drugs and liver disease:A critical review of pharmacokinetics and liver toxicity. World J Gastrointest Pharmacol Ther.

[7] Fan BE, Chong VC, Chan SS, Lim GH, Lim KG, Tan GB, Mucheli SS, Kuperan P, Ong KH (2020). Hematologic parameters in patients with COVID-19 infection. Am J Hematol.

[8] Cheng Y, Luo R, Wang K, Zhang M, Wang Z, Dong L (2020). Kidney disease is associated with in-hospital death of patients with COVID-19. Kidney Int.

[9] Bessiere F, Roccia H, Deliniere A, Charriere R, Chevalier P, Argaud L (2020). Assessment of QT Intervals in a case series of patients with coronavirus disease 2019 (COVID-19) infection treated with hydroxychloroquine alone or in combination with azithromycin in an intensive care unit. JAMA Cardiol.

[10] LaHue SC, James TC, Newman JC, Esmaili AM, Ormseth CH, Ely EW (2020). Collaborative Delirium Prevention in the Age of COVID-19. J Amer Geriatr Soc.

[11] Dubovsky A.N, Arvikar S, Stern TA, Axelrod L (2012). The neuropsychiatric complications of glucocorticoid use:steroid psychosis revisited. Psychosomatics.

[12] Vermeulen JM, van Rooijen G, van de Kerkhof MP, Sutterland AL, Correll CU, de Haan L (2019). Clozapine and long-term mortality risk in patients with schizophrenia:A systematic review and meta-analysis of studies lasting 1.1–12.5 years. Schizophr Bull.

[13] Siskind D, Honer WG, Clark S, Correll CU, Hasan A, Howes O (2020). Consensus statement on the use of clozapine during the COVID-19 pandemic. J Psychiatry Neurosci.

[14] Leung JG, Wittenberger TS, Schak KM (2020). Clozapine treated patients and COVID-19:Ensuring continued care through collaboration. Schizophr Res.

[15] Juurlink DN (2020). Safety considerations with chloroquine, hydroxychloroquine and azithromycin in the management of SARS-CoV-2 infection. Can Med Assoc J.

[16] Sanders JM, Monogue ML, Jodlowski TZ, Cutrell JB (2020). Pharmacologic treatments for coronavirus disease 2019 (COVID-19):a review. JAMA.

[17] Kunal S, Gupta K, Sharma SM, Pathak V, Mittal S, Tarke C (2020). Cardiovascular system and COVID-19:perspectives from a developing country. Monaldi Arch Chest Dis.

[18] Dong L, Hu S, Gao J (2020). Discovering drugs to treat coronavirus disease 2019 (COVID-19). Drug Discov Therapeut.

[19] Wang Y, Zhang D, Du G, Du R, Zhao J, Jin Y, Fu S, Gao L, Cheng Z, Lu Q, Hu Y (2020). Remdesivir in adults with severe COVID-19:a randomised, double-blind, placebo-controlled, multicentre trial. Lancet.

[20] Stebbing J, Phelan A, Griffin I, Tucker C, Oechsle O, Smith D (2020). COVID-19:combining antiviral and anti-inflammatory treatments. Lancet Infect Dis.

[21] Deftereos SG, Siasos G, Giannopoulos G, Vrachatis DA, Angelidis C, Giotaki SG (2020). The GReek study in the Effects of Colchicine in COvid-19 complications prevention (GRECCO-19 study):rationale and study design. Hellenic J Cardiol.

[22] Patri A, Fabbrocini G (2020). Hydroxychloroquine and ivermectin:A synergistic combination for COVID-19 chemoprophylaxis and treatment?. J Am Acad Dermatol.

[23] Sato K, Mano T, Iwata A, Toda T (2020). Neuropsychiatric adverse events of chloroquine:a real-world pharmacovigilance study using the FDA Adverse Event Reporting System (FAERS) database. Bio Sci Trends.

[24] Mohan D, Mohandas E, Rajat R (1981). Chloroquine psychosis:a chemical psychosis?. J Natl Med Assoc.

[25] Sahoo S, Kumar M, Sinha VK (2007). Chloroquine-induced recurrent psychosis. Am J Therapeut.

[26] Alisky JM, Chertkova EL, Iczkowski KA (2006). Drug interactions and pharmacogenetic reactions are the basis for chloroquine and mefloquine-induced psychosis. Med Hypoth.

[27] Akhtar S, Mukherjee S (1993). Chloroquine induced mania. Int J Psychiatry Med.

[28] de Oliveira Ribeiro NP, de Mello Schier AR, Ornelas AC, de Oliveira CM, Nardi AE, Silva AC (2013). Anxiety, depression and suicidal ideation in patients with rheumatoid arthritis in use of methotrexate, hydroxychloroquine, leflunomide and biological drugs. Compr Psychiatry.

[29] Manzo C, Gareri P, Castagna A (2017). Psychomotor agitation following treatment with hydroxychloroquine. Drug Safe Case Rep.

[30] Uthman OA, Magidson JF, Safren SA, Nachega JB (2014). Depression and adherence to antiretroviral therapy in low-, middle-and high-income countries:a systematic review and meta-analysis. Curr HIV/AIDS Rep.

[31] Wang Y, Zhang D, Du G, Du R, Zhao J, Jin Y (2020). Remdesivir in adults with severe COVID-19:a randomised, double-blind, placebo-controlled, multicentre trial. Lancet.

[32] Gupta L, Agarwal V, Ramanan AV (2020). Interleukin-6 and other cytokine blockade in COVID-19 hyperinflammation. Indian J Rheumatol.

[33] De Sousa A, Mohandas E, Javed A (2020). Psychological interventions during COVID-19:Challenges for low and middle income countries. Asian J Psychiatry.

